# Meat and Milk Inspection in Germany1Read before the Schuylkill Valley Veterinary Medical Association.

**Published:** 1895-07

**Authors:** Otto Noack

**Affiliations:** Reading, Pa.


### MEAT AND MILK INSPECTION IN GERMANY.[1]

BY OTTO NOACK, V.S.,

READING, PA.

Gentlemen: At our last meeting in Pottsville I was asked to speak of meat and milk inspection in my native country. A branch of the study of veterinary science is the law of veterinary practice. It contains two different parts; one subject to the

[1] Read before the Schuylkill Valley Veterinary Medical Association.

courts, the other to the magistrate. A part of the latter is (1) meat and (2) milk inspection.

By inspection of meat we understand the inspection by law to determine if meat is injurious to human health; secondly, the inspection of meat to be exposed for sale. This is done in order to prevent diseased or tainted meat in any form from being sold. All meat is subject to inspection in Germany, whether for private use or for the trade, with the exception of small towns or villages where there is no public slaughter-house and inspector. There only pork is subject to inspection for trichinosis. The other meats offered for sale are subject to market inspection. Where there are slaughter-houses no one is allowed to slaughter but in the slaughter-houses. Meat inspection is based on the law concerning human food, passed May 14, 1879, for the prevention of selling diseased or tainted meats To disobey this law is punishable by fine or imprisonment. Very often it is difficult to distinguish diseased meat after slaughtering, therefore an examination of cattle is made before killing, and afterward a close inspection of the intestinal organs is made. This rule must be carried out in all slaughter-houses.

The law is divided into three sections:

1. Fine for disobeying the law.
2. Explanation of what is injurious and tainted meats.
3. Inspection of meat in sick or abnormal condition.

I shall now give the legal explanation of what is meant by meat injurious to health and tainted meat. These are the principal points for the veterinary inspector to discover in the slaughter-houses, also for the inspector at the markets. Fowl, fish, and game are also subject to market inspection.

*a.* Meat is injurious to health when experience teaches that eating such meat endangers the people's health by its condition, or it is proved by science such meat is injurious to mankind.

*b.* Tainted meat is that which has such an abnormal condition that the general opinion is that this meat cannot be eaten. To this class belongs:

1. Artificial meat is any meat that is made to have the appearance of a better quality than it really is, for example, certain kinds of sausage, when they become tainted they get gray, and to make them look fresh the butcher puts on a red color by artificial means.

2. Inferior quality, when the meat comes from animals that were in a bad condition.

Now I come to the last class, as it comprises more or less of the second part.

*a*. Injurious to health, and to be condemned, is all meat coming from cattle affected with trichinosis, anthrax, glanders (the poor people eat horse-meat in different parts of the old country), hydrophobia, ulceration of the uterus, prolapsus uteri, with necrosis, calves having pyæmia or septicæmia. This also includes fowl and game.

*b*. Cysticerces of hogs and cattle is injurious to humanity, but loses all danger by boiling, but if there is a large amount of cysticerces it is condemned. The law allows it to be used after thorough boiling.

1. The fat of hogs affected with cysticerces can be used after frying or boiling.

2. To chemical preparations for soap and glue.

3. If many parts are affected the meat must be destroyed in presence of a public officer.

*c*. Meat of cattle with tuberculosis can be used, (1) providing the animal is in good condition, (2) when only one organ is affected. The inspector must act according to law, either destroy the meat or mark it as inferior quality. The parts affected are carefully destroyed, care being taken not to get into contact with other meat. When the meat is covered with pearls it is injurious to health and is condemned.

I think it is very wrong to allow any meat of tuberculous animals to be sold, if ever so little affected, as it is the most contagious and destructive disease, and only by the most rigid means can it be wiped out of existence.

*d*. Tainted (this meat can be used privately by the owner, but cannot be sold), this meat is from animals affected with:

1. Emphysema gangrænosum, erysipelas, and other infectious diseases. These diseases are not contagious to mankind, only contagious to the same class of cattle, therefore they are not injurious to human health.

2. Actinomycosis, echinococci.

3. When animals are killed too late, that they do not bleed freely.

4. Meat that has a bad odor owing to the food they eat, medicine taken, or other causes.

5. Meat of the unborn, or of animals driven or chased to death, watery or bad-looking meats.

*e*. Inferior quality, this meat is allowed to be sold for inferior quality, mentioning the circumstances why the animal was slaughtered.

The meat of animals that are sick, and not injurious to humanity, is allowed to be sold. This law includes the following:

1. Tuberculosis in low degree.

2. Pneumonia, providing no high fever or destruction of lungs and pleura; cows affected with milk fever in the first stage, tympanitis, prolapsus uteri.

3. Fractures and heavy external lesions.

4. Chronic diseases without fever, ulceration, or marasmus. Parasitic diseases of sheep are subject to the same law, providing they are killed in time.

Of what importance a good meat inspection will be for our country, according to exportation of meat, we can see by the notice of the 4th of December, 1894: The Reichsanzeiger announces that the importation into Germany of canned beef from the United States will be prohibited unless such meats are accompanied by certificates from the United States inspectors that the animals from which the meats were obtained were free from disease when slaughtered. And we never would hear such words as uttered by the Count zu Limburg Hirim on the floor of the German Parliament. The Americans demanded mild treatment of their meat and cattle exports.

Of equal importance is the examination of milk. It is subject to the same law as the meat inspection (passed May 14, 1879). The police have charge of the examination of milk. By the use of a galactometer the inspector can tell whether the milk has more or less specific weight than about 1.030 (this is the required specific weight), and must contain 13 per cent. solids. If more or less the milk is poured away. Water is most commonly used to adulterate milk—which is very easily detected by a practical eye when the milk drops down on the galactometer. After the cream is off the milk weighs heavier; to get the standard specific weight again the law allows a certain quantity of water to be added.

All cows must be examined by a veterinary surgeon. The creamery would refuse to buy otherwise. If the veterinary surgeon discovers any contagious disease the stable is closed by the police, and only by his consent can the milk be used or sold.

I have seen a case where the cows were affected with disease of feet and mouth. The attending veterinary surgeon allowed the owners to use the milk for themselves, providing they boiled it before using. Contrary to this, they used the milk unboiled. The same disease affected the entire family, showing the symptoms on gums and tongue.

It would be advisable for each county to have a veterinary surgeon employed by the Board of Health to examine the cows of farmers, especially those of dairymen. This would prevent them from selling milk from cows affected with tuberculous and other contagious diseases. This should be earnestly considered. A good reliable milk and meat inspector should be employed by the government. This would prevent us from having injurious food with germs of tubercle and other contagious diseases, and no doubt many lives would be prolonged.

